# Efficacy of midnight-noon Ebb-flow hour-prescription method combined with acupuncture at Baihui Bazhen acupoints in the rehabilitation of Aphasia after ischemic stroke

**DOI:** 10.4314/ahs.v24i4.34

**Published:** 2024-12

**Authors:** Yunbo Li, Chaoqi Lu, Lin Chen

**Affiliations:** Department of Rehabilitation Medicine, the First People's Hospital of Fuyang, Hangzhou, China

**Keywords:** Ischemic stroke, aphasia, midnight-noon ebb-flow hour-prescription method, Baihui (GV 20) Bazhen acupoints, acupuncture

## Abstract

**Background:**

To investigate the clinical effectiveness of combining the midnight-noon ebb-flow hour-prescription method with acupuncture at the Baihui (GV 20) and Bazhen acupoints for rehabilitating aphasia following ischemic stroke.

**Methodology:**

196 patients with aphasia after ischemic stroke were divided into two groups: a research group (n=98) that received language rehabilitation training along with the midnight-noon ebb-flow hour-prescription method and acupuncture at Baihui (GV 20) Bazhen acupoints, and a control group (n=98) that received only language rehabilitation training. The study recorded traditional Chinese medicine symptom scores and scores from various scales, as well as clinical efficacy.

**Results:**

The traditional Chinese medicine symptom score and National Institute of Health Stroke Scale (NIHSS) score were lower, and the scores of Stroke-Specific Quality of Life Scale (SS-QOL), Chinese Functional Communication Profile (CFCP), Montreal Cognitive Assessment (MoCA), Chinese Rehabilitation Research Center Standard Aphasia Examination (CRRCAE) were higher in the research group than in the control group after treatment. The research group also had a higher total effective rate compared to the control group.

**Conclusion:**

Midnight-noon ebb-flow hour-prescription method combined with acupuncture at Baihui (GV 20) Bazhen acupoints can effectively ameliorate NIHSS scores in patients with aphasia after ischemic stroke, facilitate language functional recovery, and improve rehabilitation.

## Introduction

Ischemic stroke is a common cerebrovascular disease in clinic and has high disability and mortality rates^1^. Aphasia, one of the common complications and sequelae of ischemic stroke, is mainly manifested as language and/or comprehension of language impairment(s), which poses serious impacts on the prognosis of patients^2^. At present, post-stroke aphasia is mostly treated by conservative therapy, in which language rehabilitation training is effective in promoting language and comprehension of language recovery and improving the prognosis of patients^3^. However, some patients' symptoms are not markedly ameliorated after rehabilitation training, since rehabilitation effects are greatly influenced by individual constitution and condition of the disease. As for midnight-noon ebb-flow hour-prescription method, appropriate acupoints are selected according to the corresponding hour. Midnight-Noon Ebb-Flow Hour-Prescription Method is a method of acupuncture treatment based on the time of the day and the time of the stem and branch to calculate the flow of qi and blood in the human body and select the corresponding Five Influence Points and the original acupuncture points. The midnight-noon ebbflow theory holds that the occurrence and progression of physical diseases are relevant to the changes in time, climates and seasons, based on which treatment regimens should be determined “according to different time and local conditions”^4^. Acupuncture therapy, which has the merits of high safety, simple operation and reasonable costs, is widely applied in the treatment of stroke sequelae and can notably reduce the effect of medication on the patient's compliance^5^. Hence, this study intends to investigate the clinical efficacy of midnight-noon ebb-flow hour-prescription method combined with acupuncture at Baihui (GV 20) Bazhen acupoints in the rehabilitation of aphasia after ischemic stroke, providing novel ideas and clinical bases for treating post-stroke aphasia.

## Patients and methods

### Patients

A total of 196 patients with aphasia after ischemic stroke treated in the Department of Rehabilitation Medicine of the First People's Hospital of Fuyang Hangzhou from January 2019 to January 2022 were included into this study. These patients were assigned to research group (n=98) and control group (n=98) using a random number table.

**Inclusion criteria involved:** 1) patients who met the diagnostic criteria for aphasia after ischemic stroke in the Guidelines for Adult Stroke Rehabilitation and Recovery: A Guideline for Healthcare Professionals From the American Heart Association/American Stroke Association^6^, 2) those aged <80 years old, 3) those with post-stroke aphasia for the first time, 4) those with clear consciousness and good compliance, and 5) those whose family members signed the informed consent and voluntarily agreed patients to participate in this study6) 7 days after the stroke.

**Exclusion criteria were as follows:** 1) patients with traumatic brain injury, 2) those with congenital coagulation disorders, 3) those with acute or chronic infection, 4) those with rib fracture or pneumothorax, 5) those with mental illness, or 6) those with other diseases potentially inducing neurological disorders. This study was approved by the Medical Ethics Committee of the First People's Hospital of Fuyang Hangzhou. The research program is in line with the Helsingin Declaration.

### Methods

Two rehabilitation assistants were trained prior to the start of the study and the two-rehabilitation assistant crossed over between the two groups of patients to implement the study protocol. The control group was administered with routine language rehabilitation training, involving 1) Cognitive training: i) Orientation: The patients were guided to speak common things' names, time and location and make self-introductions, 10-15 min/time, 2 times/day, to enhance their basic cognition and self-cognition, ii) Memory: The memory of patients was strengthened by means of chats and topics (i.e., asking them about things' names, time and location that have been learned, 10-15 min/time, 2 times/day), and iii) Calculation: The patients were guided to calculate simple mathematical problems, starting from subtraction within 10 and gradually increasing the difficulty to multiplication and division within 100, 10-15 min/time, 2 times/day. 2) Language training: i) Reading training: The patients were guided to read simple names and short sentences in children's books, 10-15 min/time, 2 times/day. Meanwhile, rehabilitation therapists should instruct patients in pronunciation and speaking speed to enhance oral expression ability, ii) Language comprehension: After describing the picture content in children's books to patients by rehabilitation therapists by means of language, the patients were guided to comprehend language and encouraged to describe details by themselves, 15-20 min/time, 2 times/day, to improve language comprehension ability.

Besides the routine language rehabilitation training administered in control group, midnight-noon ebb-flow hour-prescription method combined with acupuncture at Baihui (GV 20) Bazhen acupoints was given in research group. Midnight-noon ebb-flow hour-prescription method involved:

1. **Acupoints:** According to the principle of midnight-noon ebb-flow hour-prescription method^7^ and *Acupuncture Therapy*, Jiexi (ST 41) was selected as the main acupoint, and Huantiao (GB 30), Fengshi (GB 31), Sanyinjiao (SP 6), Zusanli (ST 36) and Taichong (LR 3) were selected as the matching acupoints.
2. **Procedures:** The patients were instructed to sit upright, with body and mind relaxed and eyes facing forward. After the acupoints were disinfected routinely, the acupuncture was operated between 10:00 and 11:00 every day. Specifically, Jiexi (ST 41) was pricked using sterile acupuncture needles (0.25 mm × 40 mm, Hwato) by tonifying method, while acupoints such as Huantiao (GB 30), Fengshi (GB 31), Sanyinjiao (SP 6), Zusanli (ST 36) and Taichong (LR 3) were pricked by warming-dredging needling method. The needles were retained for 20-30 min after obtaining qi, and the pinholes were pressed with sterile/sterilized cotton swabs when needles were withdrawn. This acupuncture therapy was given once daily, 5 times/week, 4 weeks for a course of treatment. The acupuncture at Baihui (GV 20) Bazhen acupoints was operated as follows: 1) Acupoints: The patients were in the supine position, and then a circle was drawn with Baihui (GV 20) as the center and the distance from Yintang (EX-HN 3) to Baihui (GV 20) as the radius. The circle was divided into 8 areas, serving as Bazhen. The 1/8 area just above the circumference was Tiangan, and counterclockwise directional areas corresponded to Duihu, Liniao, /span>Longzhen, Dikun, Yungen, Shekan, and Fengxun. 2) Procedures: The patients remained in the supine position. After the areas used for acupuncture were routinely disinfected, the acupoints were pricked using sterile acupuncture needles (0.25 mm × 40 mm, Hwato), with an angle of 15 degrees to the scalp and a depth of 25 mm. All acupuncture procedures at Bazhen acupoints were carried out by lifting, thrusting and twirling methods. The needles were retained for 20-30 min after obtaining qi. This acupuncture therapy was given once daily, with an interval of 1 day, 3 times/week, 4 weeks for a course of treatment.

### Location of the acupoints

Jiexi (ST 41) belongs to the foot Yangming Stomach meridian. Meridian (fire) point. It is located in the central depression of the transverse stripe at the junction of the dorsum of the foot and the lower leg, between the long extensor tendon of the thumb and the long extensor tendon of the toe. The superficial peroneal nerve, the deep peroneal nerve and the anterior tibial artery and vein are located there. Huantiao (GB 30) is a meridian point of the foot Shaoyang gall bladder meridian. It is near the hip joint and is responsible for lower limb movements, referring to when the lower limb bends the knee and flexes the hip in a circular jump. This point can be touched by the heel of the foot. Fengshi (GB 31) is on the midline of the lateral thigh, 7 inches above the level of the transverse popliteal stripe, and is easily located by standing upright with the hand down at the side of the body and the tip of the middle finger. Sanyinjiao(SP 6) is on the inner side of the calf, 3 inches above the tip of the inner ankle, at the posterior border of the medial edge of the tibia. Zusanli (ST 36) is located in the depression below the lateral kneecap of the leg about 4 fingers wide downwards. Taichong (LR 3) is on the back of the foot, between the 1st and 2nd metatarsal bones, in the depression anterior to the metatarsal tuberosity, or where the artery fluctuates when touched. Baihui (GV 20) is at the intersection of the midline at the top of the head and the line joining the tips of the two ears. Yintang (EX-HN 3) is on the forehead, at the intersection of the line between the two eyebrows and the front median line.

### Observational indicators

The traditional Chinese medicine symptom score and scores of Stroke-Specific Quality of Life Scale (SS-QOL)^8^, Chinese Functional Communication Profile (CFCP)^9^, Montreal Cognitive Assessment (MoCA)^10^, Chinese Rehabilitation Research Center Standard Aphasia Examination (CRRCAE)^11^ and National Institute of Health Stroke Scale (NIHSS)^12^ as well as clinical efficacy were compared between the two groups. The scoring criteria were as follows: 1) Traditional Chinese medicine symptom score: The symptoms (i.e., sluggish or no speech, loss or disappearance of sensation and perception, and deviation of corner of the mouth and tongue) were scored 0-4 points according to the severity (no → severe), and the scores of both groups were counted. 2) Neurological function and quality of life: Before and after treatment, the NIHSS scores (totally 42 points) were recorded, with a higher score corresponding to more serious neurological impairment, and the SS-QOL scores (totally 49 items) were recorded, which had positive relations with the quality of life. 3) Language function: The scores of CFCP (totally 250 points), MoCA (totally 30 points) and CRRCAE (totally 72 points) were recorded before and after treatment. The CFCP and CRRCAE scores were positively related to the language ability of patients with post-stroke aphasia, while the MoCA score was positively correlated with the cognitive ability of patients with post-stroke aphasia. 4) Clinical efficacy: At 4 weeks after treatment, the therapeutic effects of the two groups were evaluated with the following criteria^6^,^7^: i) Remarkably effective: The patient's language abilities including listening, speaking, reading and writing were markedly improved and basic self-care could be performed after treatment, with a decrease of >70% in the total traditional Chinese medicine symptom score, ii) Effective: The patient's language abilities including listening, speaking, reading and writing were improved after treatment, with a decrease of 50-70% in the total traditional Chinese medicine symptom score, and iii) Ineffective: The patient's condition did not meet the criteria of “remarkably effective” and “effective”. The total effective rate = number of (remarkably effective case + effective case)/total case.

### Statistical analysis

Statistical Product and Service Solutions (SPSS) 22.0 software (IBM, Armonk, NY, USA) was adopted for data analysis. Enumeration data (i.e., gender, educational level, and clinical efficacy) were analysed by y2 test, and measurement data (i.e., age, course of disease, symptom score, SS-QOL score, CFCP score, MoCA score, CRRCAE score, and NIHSS score) were analysed by t-test. P<0.05 was considered statistically significant.

## Results

### Comparison of general data between the two groups

There was no significant difference in age, stroke duration and aphasia duration between the two groups (*t* = 0.801, 1.261, 0.604, *P* > 0.05), but there was no significant difference in education level and gender (x^2^ = 0.259, 0.898, *P* > 0.05), as shown in Table 1.

**Table 1:** Comparison of general data between the two groups

Group	N	Age (years)	Stroke duration (months)	Duration of aphasia (d)	Education level [n, (%)]			Gender	
	
					Primary school or below	High school	University and above	Male	Female
Study group	98	60.35 ± 4.15	6.41 ± 1.12	12.35 ± 2.15	45 (45.92)	40 (40.82)	13 (13.27)	58 (59.18)	40 (40.82)
Control group	98	59.89 ± 3.89	6.68 ± 1.80	12.17 ± 2.02	42 (42.86)	41 (41.84)	15 (15.31)	46 (46.94)	42 (42.86)
*t/x^2^*		0.801	1.261	0.604	0.259			0.898	
*P*		0.424	0.209	0.547	0.879			0.343	

### Comparison of TCM symptom scores between the two groups

Before treatment, there was no significant difference in scores of speech obscurity or aphasia, subsidence or disappearance of sensory perception, and deviation of mouth and tongue between the two groups (*t* = 0.699, 1.428, 0.800, *P* > 0.05); after treatment, scores of speech obscurity or aphasia, subsidence or disappearance of sensory perception, and deviation of mouth and tongue in the study group were lower than those in the control group (*t* = 21.779, 24.418, 16.740, *P* < 0.05), as shown in Table 2.

**Table 2:** Symptom score comparison between the two groups (x̅±s, points)

Group	N	Speech obscurity or aphasia		Perception subsided or disappeared		Deviation of mouth and tongue	

		Before treatment	After treatment	Before treatment	After treatment	Before treatment	After treatment
Control group	98	3.20 ± 0.21	1.15 ± 0.12	3.08 ± 0.24	1.05 ± 0.09	2.91 ± 0.18	0.89 ± 0.08
Study group	98	3.22 ± 0.19	1.59 ± 0.16	3.03 ± 0.25	1.44 ± 0.13	2.89 ± 0.17	1.12 ± 0.11
*t*		0.699	2 1.779	1.428	2 4.418	0.800	1 6.740
*P*		0.485	<0.001	0.155	<0.001	0.425	<0.001

### Comparison of neurological function and quality of life between the two groups

Before treatment, there was no significant difference in SS-QOL and NIHSS between the two groups (*t* = 0.284, 0.226, *P* > 0.05); after treatment, SS-QOL was higher and NIHSS was lower in the study group than in the control group (*t* = 6.846, 7.582, *P* < 0.05), as shown in Table 3.

**Table 3:** Comparison of neurological function and quality of life between the two groups (x̅±s, points)

Group	N	S S-QOL		N IHSS	

		Before treatment	After treatment	Before treatment	After treatment
Control group	98	95.10 ± 13.79	165.45 ± 20.43	12.42 ± 2.20	8.43 ± 1.85
Study group	98	94.53 ± 14.35	147.79 ± 15.32	12.35 ± 2.13	6.72 ± 1.25
*t*		0.284	6.846	0.226	7.582
*P*		0.777	<0.001	0.821	<0.001

### Language function comparison between the two groups

Before treatment, there was no significant difference in C FCP, Mo CA and CRRCAE scores between the two groups (*t* = 0.809, 0.378, 0.175, *P* > 0.05); after treatment, C FCP, Mo CA, and CRRCAE scores were higher in the study group than in the control group (*t* = 19.670, 7.665, 6.516, *P* < 0.05) as shown in Table 4.

**Table 4:** Comparison of language function between the two groups (x̅±s, points)

Group	N	CFCP		MoCA		CRRCAE	

		Before treatment	After treatment	Before treatment	After treatment	Before treatment	After treatment
Control group	98	83.35 ± 7.62	169.97 ± 8.86	19.68 ± 2.43	26.63 ± 3.15	25.53 ± 3.46	58.86 ± 4.32
Study group	98	82.46 ± 7.79	144.62 ± 9.18	19.55 ± 2.39	23.32 ± 2.89	25.47 ± 3.37	54.41 ± 5.20
*t*		0.809	19.670	0.378	7.665	0.175	6.516
*P*		0.420	<0.001	0.706	<0.001	0.862	<0.001

### Comparison of clinical efficacy between the two groups

The overall response rate in the study group (9 4.90% vs 85.71%) was higher than that in the control group (x^2^ = 4.721, *P* < 0.05), as shown in Table 5.

**Table 5:** Comparison of clinical efficacy between the two groups [n (%)]

Group	N	Markedly effective	Effective	Invalid	Overall response rate
Control group	98	61 (62.25)	32 (32.65)	5 (5.10)	93 (94.90)
Study group	98	51 (52.04)	33 (33.67)	14 (14.29)	84 (85.71)
*X* ^2^					4.721
*P*					0.030

## Discussion

Strokes are a significant health concern in China, with over 12 million stroke patients reported in 2018, causing over 1.9 million deaths annually and leading to disability. Strokes can cause severe neurological and motor impairment, reducing the quality of life of patients^13^. As a result, early and targeted treatment is necessary to improve patient conditions and reduce the treatment burden. Aphasia, a common consequence of stroke, affects 20-45% of stroke patients, impairing their ability to function in daily life, and increasing negative emotions like low self-esteem and depression, which hinders physical recovery^14^. Currently, language rehabilitation training is the primary clinical treatment for post-stroke aphasia, but traditional language training is often long and monotonous, and effectiveness varies widely among patients. Repeated language training can aid in nerve conduction pathway reconstruction, restoring language function and improving clinical symptoms.

In traditional Chinese medicine, ischemic stroke is classified into “stroke”, and its primary pathogenesis is considered the brain vessel obstruction resulting from qi and blood disorders. In recent years, as traditional Chinese medicine therapies such as acupuncture and massage are gradually applied in the rehabilitation of stroke sequelae, traditional Chinese medicine therapies have been recognized to be effective and safe in treating cerebrovascular complications^15^. In this study, midnight-noon ebb-flow hour-prescription method combined with acupuncture at Baihui (GV 20) Bazhen acupoints was used in the treatment of aphasia after ischemic stroke. In the midnight-noon ebb-flow hour-prescription method, the common acupoints for treating stroke, such as Jiexi (ST 41), Huantiao (GB 30), Fengshi (GB 31) and Sanyinjiao (SP 6), were selected based on the condition characteristics of patients with ischemic stroke. Moreover, the operation was conducted between 10:00 and 11:00 every day according to the principle of midnight-noon ebb-flow hour-prescription method. Acting on Jiexi (ST 41) is capable of calming fright, relaxing sinews, activating collaterals, and tranquilizing mind. Acting on Huantiao (GB 30) is able to strengthen waist and knees and dispel wind and dampness. After being stimulated, Zusanli (ST 36) has the effects of reinforcing healthy qi and eliminating pathogenic qi, dredging channels and activating collaterals, and regulating the spleen and stomach. The application of midnight-noon ebb-flow hour-prescription method can effectively relieve the local blood circulation of patients with post-stroke aphasia, and promote the functional recovery of language central nerve of patients^16^. Additionally, the acupuncture at Baihui (GV 20) Bazhen acupoints has favourable effects of regulating qi and unblocking collaterals, and replenishing qi and raising yang, which, in combination with midnight-noon ebb-flow hour-prescription method, exerts the effects of relaxing sinew and activating collaterals as well as calming fright and tranquilizing mind, thus improving cerebral blood flow perfusion, facilitating language functional recovery, and improving the rehabilitation effect of patients with post-stroke aphasia^17^. This article still has several limitations. For example, this study was only conducted on patients with aphasia after ischemic stroke, and patients diagnosed with post-stroke aphasia for the first time. Besides, the sample size included in this study was small and this study was a single-centre study.

## Conclusion

The efficacy of the Wu Liu Chui Na Zi method combined with acupuncture at the Bai Hui eight acupoints in the treatment of post-ischaemic stroke aphasia can effectively improve the NIHSS scores of patients with post-ischaemic stroke aphasia, promote the recovery of patients' language function and improve the rehabilitation treatment effect of patients, and has clinical promotion value.

## Data Availability

The datasets used and analysed during the current study are available from the corresponding author on reasonable request.

## References

[1] Chen L, Wang W, Zhang S, Liu H, Yuan X, Yang X (2021). Value of Barthel, PLAN and NIHSS scores for predicting the death of patients with acute ischemic stroke during their 5-year follow-up. J Clin Neurosci.

[2] Barrows PD, Thomas SA, Van Gordon W (2021). Assessing Self-Reported Mood in Aphasia Following Stroke: Challenges, Innovations and Future Directions. J Stroke Cerebrovasc.

[3] Zumbansen A, Black S E, Chen JL (2020). P240 Comparing the effectiveness of rTMS and tDCS for aphasia recovery after stroke. Clinical Neurophysiology.

[4] Li Zhengjie Z, Jun C (2020). Cerebral fractional amplitude of low-frequency fluctuations may predict headache intensity improvement following acupuncture treatment in migraine patients. Journal of Traditional Chinese Medicine.

[5] You Y, Zhang T, Shu S, Qian X, Zhou S, Yao F (2020). Wrist-ankle acupuncture and Fluoxetine in the treatment of post-stroke depression: a randomized controlled clinical trial. J Tradit Chin Med.

[6] Winstein CJ, Stein J, Arena R, Bates B, Cherney LR, Cramer SC (2016). Guidelines for Adult Stroke Rehabilitation and Recovery: A Guideline for Healthcare Professionals from the American Heart Association/American Stroke Association. Stroke.

[7] Zhang SJ (2015). [On Ziwuliuzhu (midnight-noon ebb-flow) needling of acupuncture theory and rigidification process of acupuncture theory in the Jin and Yuan Dynasties]. Zhen Ci Yan Jiu.

[8] Ozen S, Senlikci HB, Guzel S, Yemisci OU (2021). Computer Game Assisted Task Specific Exercises in the Treatment of Motor and Cognitive Function and Quality of Life in Stroke: A Randomized Control Study. J Stroke Cerebrovasc.

[9] Li J, Hong L, Bi HY, Yang Y (2021). Functional brain networks underlying automatic and controlled handwriting in Chinese. Brain Lang.

[10] Gulke E, Alsalem M, Kirsten M, Vettorazzi E, Choe CU, Hidding U (2022). Comparison of Montreal cognitive assessment and Mattis dementia rating scale in the preoperative evaluation of subthalamic stimulation in Parkinson's disease. Plos One.

[11] Kong AP, Chan KP, Jagoe C (2021). Systematic Review of Training Communication Partners of Chinese-speaking Persons with Aphasia. Arch Rehabil Res Clin Transl.

[12] Zeitlberger AM, Flynn MC, Hollenstein M, Hundsberger T (2021). Assessment of neurological function using the National Institute of Health Stroke Scale in patients with gliomas. Neuro-Oncol Pract.

[13] Qin H, Chen Y, Liu G, Turnbull I, Zhang R, Li Z (2021). Management characteristics and prognosis after stroke in China: findings from a large nationwide stroke registry. Stroke Vasc Neurol.

[14] Leff AP, Nightingale S, Gooding B, Rutter J, Craven N, Peart M (2021). Clinical Effectiveness of the Queen Square Intensive Comprehensive Aphasia Service for Patients with Poststroke Aphasia. Stroke.

[15] Yang J (2021). Acupuncture treatment for post-stroke insomnia: A systematic review and meta-analysis of randomized controlled trials. Complement Ther Clin.

[16] Zhang Y, Wang Z, Jiang X, Lv Z, Wang L, Lu L (2021). Effectiveness of Acupuncture for Poststroke Aphasia: A Systematic Review and Meta-Analysis of Randomized Controlled Trials. Complement Med Res.

[17] Liu J, Liu Y C, Zhi-Hui M A (2018). Parallel Controlled Study of Needling Different Acupoints Combined with Schuell Language Training in the Treatment of Aphasia after Ischemic Stroke. Journal of Clinical Acupuncture and Moxibustion.

